# *Pseudogymnoascus destructans* invasion stage impacts the skin microbial functions of highly vulnerable *Myotis lucifugus*

**DOI:** 10.1093/femsec/fiae138

**Published:** 2024-10-14

**Authors:** Virginie Lemieux-Labonté, Jananan S Pathmanathan, Yves Terrat, Nicolas Tromas, Anouk Simard, Catherine G Haase, Cori L Lausen, Craig K R Willis, François-Joseph Lapointe

**Affiliations:** Département de sciences biologiques, Université de Montréal, Montréal, Québec, H2V 0B3, Canada; Institut de Systématique, Evolution, Biodiversité (ISYEB), Sorbonne Université, Paris, 75005, France; Département de sciences biologiques, Université de Montréal, Montréal, Québec, H2V 0B3, Canada; Département de sciences biologiques, Université de Montréal, Montréal, Québec, H2V 0B3, Canada; Ministère de l’Environnement, de la Lutte contre les changements climatiques, de la Faune et des Parcs, Québec, G1R 5V7, Canada; Department of Biology, Austin Peay State University, Clarksville, TN, 37044, United States; Wildlife Conservation Society Canada, Kaslo, British-Columbia, V0G 1M0, Canada; Department of Biology and Centre for Forest Interdisciplinary Research, University of Winnipeg, Winnipeg, Manitoba, R3B 2E9, Canada; Département de sciences biologiques, Université de Montréal, Montréal, Québec, H2V 0B3, Canada

**Keywords:** microbial function assemblage, shotgun metagenomic, skin microbiome, *Pseudogymnoascus destructans*, white-nose syndrome, *Myotis lucifugus*, little brown bats

## Abstract

The role of the skin microbiome in resistance and susceptibility of wildlife to fungal pathogens has been examined from a taxonomic perspective but skin microbial function, in the context of fungal infection, has yet to be studied. Our objective was to understand effects of a bat fungal pathogen site infection status and course of invasion on skin microbial function. We sampled seven hibernating colonies of *Myotis lucifugus* covering three-time points over the course of *Pseudogymnoascus destructans* (Pd) invasion and white nose syndrome (pre-invasion, epidemic, and established). Our results support three new hypotheses about Pd and skin functional microbiome: (1) there is an important effect of Pd invasion stage, especially at the epidemic stage; (2) disruption by the fungus at the epidemic stage could decrease anti-fungal functions with potential negative effects on the microbiome and bat health; (3) the collection site might have a larger influence on microbiomes at the pre-invasion stage rather than at epidemic and established stages. Future studies with larger sample sizes and using meta-omics approaches will help confirm these hypotheses, and determine the influence of the microbiome on wildlife survival to fungal disease.

## Teaser

*Myotis lucifugus* microbiome profiles change in the epidemic stage of *Pseudogymnoascus destructans* infvasion but microbiome could reestablished over time.

## Introduction

Fungal diseases are on the rise worldwide, causing unprecedented declines in wildlife populations (Daszak 2000, Jones et al. 2008, Fisher et al. 2012, 2020). The emergence of fungal skin infections with physiological consequences is of particular concern as they raise new challenges in conservation biology, operating by mechanisms not yet fully characterized (Fischer et al. 2020). Chytridiomycosis, caused by the fungus *Batrachochytrium dendrobatidis* (Berger et al. 1998), has caused decline in at least 501 amphibian species (6.5% of described amphibian species), 18% of which are confirmed or presumed extinct and 25% of which have experienced declines in abundance exceeding 90% (Scheele et al. 2019). Similarly, *Ophidiomyces ophidiicola*, the fungal agent of ophidiomycosis, is a potential threat to the European snake population and has potentially caused declines in North America (Allender et al. 2011, 2015, Clark et al. 2011, Franklinos et al. 2017).

The fungus *Pseudogymnoascus destructans* (Pd) (Gargas et al. 2009, Lorch et al. 2011), pathogenic agent of white-nose syndrome (WNS), has impacted North American bats over the last decade (Blehert et al. 2009, Frick et al. 2010, Frick et al. 2016). Pd invades the skin during hibernation, forming lesions that alter fluid balance, thermoregulation, and gas exchange, resulting in increased hibernal arousal frequency (Meteyer et al. 2009, Cryan et al. 2010, Warnecke et al. 2013, Verant et al. 2014, McGuire et al. 2017). Three species, northern myotis bats (*Myotis septentrionalis*), little brown bats (*Myotis lucifugus*), and tricolored bats (*Perimyotis subflavus*) declined by >90% since WNS arrival in North America (Cheng et al. 2021). In Canada, these species are listed as federally endangered due to mortality rates of 75%–90% during the several-year invasion stage of the disease (Canadian Wildlife Service 2014). Despite dramatic declines during the invasion and epidemic stages of the disease, some hibernating colonies of *M. lucifugus* have persisted following fungal invasion (Dobony et al. 2011, Langwig et al. 2012; Canadian Wildlife Service 2014; Frick et al. 2015, Vanderwolf and McAlpine 2021, Hooton et al. 2023), with population counts stabilizing at ∼5%–30% of their initial sizes (Langwig et al. 2012, Frick et al. 2015). Variation in susceptibility to Pd could reflect mechanisms that allow for resistance (i.e. reduction or elimination of pathogen infection) and/or tolerance (i.e. reduction of the damage caused by infection) (Råberg et al. 2007, Råberg et al. 2009, Svensson and Råberg 2010). WNS resistance and tolerance are increasingly being studied, with mechanisms ranging from immune system response, increase in fat accumulation, roost selection, torpor regulation, and skin microbiome response (Hoyt et al. 2015, Frick et al. 2016, Langwig et al. 2017, Lemieux-Labonté et al. 2017, Lilley et al. 2017, Cheng et al. 2019, Auteri and Knowles 2020).

In a fungal disease context, the microbiome could play an important role in species’ resistance or vulnerability. Originally, the microbiome has been defined as a collection of microorganisms (bacteria, archaea, eukaryotes, and viruses), their genomes (i.e. genes), and surrounding environmental conditions (Marchesi and Ravel 2015). However, most microbiome studies only investigated bacteria and/or, to some extent, fungi. Archaea, other eukaryotes, and viruses have been poorly characterized in comparison to these two taxonomic groups, in particular due to limited methods and resources. Therefore, when referring to microbiome studies in this paper, we will mostly be referring to the bacterial and/or fungal component of the microbiome.

The microbiome could be a critical component of host health, influencing biochemical and physiological processes, including defense against pathogens (Cho and Blaser 2012). The microbiome contributes to host defense by limiting colonization and persistence of pathogens that compete for resources, and by producing pathogen inhibitors (Grice and Segre 2011, Walter et al. 2018, Woo et al. 2019). It also interacts with the innate and adaptive immune systems, and contributes to the maintenance of skin integrity and tissue repair (Lai et al. 2009, Curtis and Sperandio 2011, Naik et al. 2012). Several studies have highlighted the potential role of the skin microbiome in the resistance and susceptibility to multiple fungal pathogens (Harris et al. 2009, Bletz et al. 2013, 2017, Jani and Briggs 2014, Woodhams et al. 2014, Bataille et al. 2016, Lemieux-Labonté et al. 2017, 2020; Rebollar et al. 2019, Ange-Stark et al. 2023). However, previous studies have largely focused on the taxonomic composition of bacterial communities to infer microbiome functions.

Based on taxonomical analysis obtained by amplicon sequencing, the bat microbiome, contrary to other mammals, is mostly influenced by ecological factors, and not by phylogenetic relationships (Lutz et al. 2019). Microbial community assemblage of gregarious bats changes over time at the colony level (Kolodny et al. 2019), and is predominantly influenced by the environment (Avena et al. 2016, Lemieux-Labonté et al. 2016, Winter et al. 2017, Grisnik et al. 2020). In accordance with a protective effect of the skin microbiome, hundreds of microorganisms isolated from wild bats and their corresponding habitats have shown inhibitory effects on Pd by secreted compounds, contact inhibition, or volatile molecules (Hamm et al. 2017, Micalizzi et al. 2017). Additionally, compared with the microbiome of the cave environment, the bat microbiome is enriched with antifungal taxa (Grisnik et al. 2020). Indeed, antifungal strains of *Pseudomonas* isolated from the skin of *E. fuscus* inhibit Pd growth *in vitro* (Hoyt et al. 2015, Hamm et al. 2017), and improve survival of WNS-affected little brown bats when applied to the skin as a probiotic treatment *in vivo* (Cheng et al. 2017, Hoyt et al. 2019). The survival of *M. lucifugus* may be due to commensal bacteria, as persisting colonies with Pd exhibit a less diverse microbiome, and proportionally more antifungal bacterial taxa on their skin (Lemieux-Labonté et al. 2017). Accordingly, with the hypothesis that the microbiome protects against Pd, Grisnik et al. (2020) found that Pd-negative bats exhibited microbial assemblages with more antifungal taxa than Pd-positive bats in *P. subflavus*. Moreover, one study has observed a significant reduction of bacterial community richness and evenness after invasion by Pd in *M. lucifugus* (Ange-Stark et al. 2023). These changes could be indicative of bacterial selection, but they could also be indicative of altered microbiome ability to perform functions that inhibit or prevent the growth of pathogens and/or opportunistic bacteria, and may in turn potentially amplify disease severity. Taken together, these findings show that the response of host microbial communities with respect to fungal pathogens is still unclear.

While amplicon sequencing can provide information about the effects of Pd on the taxonomic composition of the microbiome, we still know little about its effects on microbiome functions. In *P. subflavus*, Grisnik et al. (2022) reported different patterns for taxonomical versus functional assemblages. While taxon-based analysis revealed differences between Pd-positive and Pd-negative bats, functional assemblages remained unchanged by Pd-infection (Grisnik et al. 2022). Other recent work suggests that functional characterization of the microbial community is more sensitive than taxonomic assessment, and that genes, rather than species or genera, may be the appropriate parameter for understanding patterns of diversity in many microbial communities (Green et al. 2008, Burke et al. 2011, Louca et al. 2016, Ma et al. 2019). Furthermore, metagenomics yield greater resolution and accuracy in detection of putative functional genes compared to amplicon sequencing (Poretsky et al. 2014, Louca et al. 2016, Ranjan et al. 2016, Brumfield et al. 2020, Durazzi et al. 2021). Metagenomics should thus be used to study microbiomes in relation to taxonomy and/or functions (genes) (Langille 2018), and functional investigation is required to unravel the role of microbiome in vulnerable bat species facing WNS.

Previous WNS studies have investigated bat microbiomes based only on positive or negative individual Pd status. For example, Grisnik et al. (2022) found no difference in microbiome functions between Pd-positive and Pd-negative *P. subflavus*. However, responses of the microbiome to Pd could also change over the course of disease invasion. Following initial invasion when Pd prevalence is low but increasing, WNS, like many pathogen outbreaks, is characterized by an epidemic phase during which the host population declines and pathogen prevalence is moderate to high (Langwig et al. 2015a). Following the epidemic phase, Pd appears to become established with, for *M. lucifugus*, high and stable prevalence and stable (though smaller than original) host population size (Langwig et al. 2012, 2015a, Frick et al. 2015). Understanding how Pd infection status and disease stage interact with skin microbial function assemblage is important for understanding mechanisms underlying persistence of bats.

We assessed the functional potential of the skin microbiome of wild hibernating *M. lucifugus* colonies using shotgun metagenomics. We compared bats from colonies at three stages of the WNS outbreak (Pd-negative/pre-invasion; Pd-positive/epidemic; Pd-positive/established) (Langwig et al. 2015b) to (1) disentangle effects of site infection status (i.e. Pd-positive vs. Pd-negative) from the stage of pathogen invasion on the gene functions in the microbiome and (2) identify differences in microbial functions that could affect, or be affected, by Pd. Based on previous results in *M. lucifugus* (Lemieux-Labonté et al. 2017, Ange-Stark et al. 2023), we hypothesized that Pd would affect this species microbiome functional assemblage, with a decrease in functional diversity during the epidemic phase of rapid population decline. This reduced diversity will persist into the established phase with stable populations of persisting, potentially resistant or tolerant bats. We also hypothesized that genes associated with antifungal properties, or which act in competition with fungi, would be more prevalent in persisting bats following Pd establishment.

## Materials and methods

### Skin microbiome collection

We sampled skin microbiome from *M. lucifugus* in seven different hibernacula from 2015 to 2019 that were in either the pre-invasion, epidemic (one year after first Pd detection and observed population decline) or established phases (9 years after first Pd detection and noticeable WNS decline with actual stable population trend) of WNS. We sampled one of the seven sites longitudinally during both the pre-invasion and epidemic stages. We collected samples from 10 to 15 bats and one negative field control (humified swab open in the air) per hibernaculum. All methods were approved by the Animal Welfare and Ethics Committee at Université de Montréal (Protocol Number #16-015), Montana Fish, Wildlife and Park (Permit Number #2018-008-W), and the University of Winnipeg Animal Care Committee (Protocol Number AEO5639). We used two different protocols depending on the site, as the sites were part of different monitoring programs. We controlled for these procedural differences in our statistical analysis. For the first method (M1), bats were swabbed in the hand because samples also needed to be collected for other purposes. Bats were captured by gently pulling them off the wall and microbiome samples were collected while handling the bats using a clean pair of exam gloves for each bat. We swabbed the dorsal side of the right wing (forearm and wing skin) in overlapping linear strokes for 12 s with a sterile Puritan HydraFlock swab (Fisher Scientific) soaked in sterile 0.15 M NaCl 0.1% Tween-20 buffer. Swab tips were ejected into a 1.5-ml microtube containing the same buffer. Tubes were transferred to 4°C within 2 hr and to −20°C within 24 hr of sampling (see Table 1 for more information on the samples). All samples were shipped to Université de Montréal (Québec, Canada) for further processing. This method was used at one hibernaculum located 60 km south of Great Falls, Montana, USA (47°30′N, -111°1′W), and a second hibernaculum located in Alberta, Canada (53°5′N, 116°34′W). Both hibernacula were in the pre-invasion stage and Pd-negative at time of collection in 2018 (geographically isolated hibernacula from known positive sites and no decline observed at sampling time). Twenty bats (10 per site) were collected using this method. The Montana samples were heated to 56°C for 30 min to ensure eradication of potential rabies viruses prior to being shipped on dry ice, as requested by USDA animal products export certificates 0579–0256 and Canadian Food Inspection Agency importation permit A-2017–06316–4.

**Table 1.** Pooled bat skin microbiome samples information collected at different sites for different Pd invasion stages.

Site Pd status	Invasion stage	Site	Province/State	Hibernaculum	Rock	Number of bats per site	Number of pools	Months of collection	Collection years^1^	Collection method^2^
Negative	Pre-invasion	Alberta	Alberta	Cave	Limestone	10	2	January	Early 2018	M1
								September	Late 2019	
		Montana	Montana	Cave	Limestone	10	2	February	Late 2018	
		Patate	Québec	Cave	Limestone	10	2	November	Early 2015	M2
		St-George (SG)	Ontario	Cave	Limestone	5	1	February	Late 2016	
Positive	Epidemic	Richard Lake	Ontario	Mine	Granite	15	3	March	Late 2018	
		St-George (SG)	Ontario	Cave	Limestone	10	2	April	Late 2019	
	Established	Lafleche	Québec	Cave	Calcite	10	2	February	Late 2019	
		Lames	Québec	Cave	Calcite	10	2	February	Late 2019	

1 Late indicates samples collected in late winter (January to April) and early indicates samples collected in early winter (September to November).

2 Method M1: bats were handled and swabbed 12 s on the right wing. Method M2: bats were swabbed 20 s on back and wing while hanging on roost wall. Each pool is a mixed of five individuals collected at the same site.

We used a slightly different sampling protocol (M2) for the other five hibernacula, as samples were collected as part of annual visual survey where bats were not handled. For these sites, we randomly selected hibernating bats from those we could reach from the ground. Samples were collected without handling the bats by swabbing the back (fur) and wing forearm (skin) of each bat in overlapping linear strokes for 20 s with a sterile Whatman Omniswab (Fisher Scientific) soaked in sterile 0.15 M NaCl. We swabbed these bats longer as swabbing was harder to perform while not handling the bats to ensure quantities of biomass collected are similar to M1 methods. Swab tips were ejected into MoBio Powersoil DNA isolation Kit tubes (MoBio Laboratories), which were transferred to −20°C within 24 h of sampling. We used this method at one site (Patate, pre-invasion) on Anticosti island, Québec, Canada (49°29′N, −63°00′W), one site near Lake St-George (SG, 2016/pre-invasion and 2019/epidemic), Manitoba, Canada (51°22′N, 97°14′W), one site at Richard Lake (RL, epidemic) ∼100 km east of Kenora, Ontario, Canada (49°45′N, 94°28′W), and two sites (Lames and Laflèche, established) located 60 km north of Gatineau, Québec, Canada (45°28′N, 75°42′W). Sixty bats were collected with this method and used for analysis. Pd site status was determined at epidemic sites by collecting separate swabs and performing qPCR (Table S0). For established sites, multiple qPCR assessment has been performed years before and after sampling, and they showed positive results for qPCR (Table S0 shows results for 2020, i.e. one year from microbiome collection). All samples with values crossing baseline below 40 cycles threshold (40 C_t_) were considered positive. Mass bat mortalities were recorded in newly Pd-positive sites indicating that colonies were in the Pd epidemic invasion stage. In Pd-established sites, bat mortality rates were stable and similar to pre-invasion rates. The collection years indicate whether the samples were collected in early or late winter of the corresponding year.

### DNA extraction, purification and metagenomic sequencing

Bacterial genomic DNA was extracted from each swab using the ChargeSwitch gDNA Mini Bacteria Kit (Invitrogen^TM^). To increase DNA yield, we modified the manufacturer's protocol by adding a 15-min heating period and eluting in 50 µl of elution buffer. All procedures were conducted with gloves in a laminar flow hood to limit sample contamination, and extractions were randomized to avoid detecting false patterns (Salter et al. 2014). Because of low input DNA and the fact that bat microbiomes show low inter-individual variation within colonies (Kolodny et al. 2019), samples were pooled by sites and collection years. Samples were randomly pooled within site. Therefore, each line in Table 1 represents a pooled sample from five individuals occupying the same site. Pooled samples were purified using a purification kit spin column (Zymo Research^TM^), and sample concentrations were measured with a Qubit 2.0 Fluorometer (Invitrogen). Samples with DNA concentration below 0.01 ng/µl were discarded (including field negative control), except for extraction controls that were used to keep track of contamination. To improve quality, we added a mix of 20 µl nuclease-free water, 51 µl NEB3 buffer, and 3 µl RNase to each sample prior to heating at 37°C for 60 min, followed by 70°C for 20 min. Samples were then sent to Génome Québec facilities at McGill University (Québec, Canada) for shotgun DNA library preparation with NEB Ultra II kit (New England BioLabs Inc.). Ten samples failing the quality control test were discarded, including all three pooled extraction controls. Metagenomic DNA sequencing was performed at Génome Québec facilities on NovaSeq6000 S4 flow cells of type 150 bp paired-end (Illumina).

### Sequence processing

We obtained a total of 320 713 730 shotgun sequence reads and 96 855 546 460 bases for the 16 bat sample pools under study (Table S1 for details on the raw sequences). No sequences were obtained from extraction negative controls. Sequence reads were mapped to the *M. lucifugus* genome, and only unmapped reads representing bat skin microbial genes (i.e. genes from other eukaryotes, prokaryotes, and viruses) were selected for further analysis. First, quality trimming of raw reads was performed using the SolexaQA v3.1.7.1 program (Cox et al. 2010) with default parameters. Trimmed reads shorter than 75 nt were removed for further analysis. Reads with identical leading 20 bp were removed as artificial duplicates. From the trimmed high-quality reads, gene fragments were predicted using FragGeneScan-Plus v3.0 (Kim et al. 2015). Predicted protein fragments were clustered at a 90% similarity level using cd-hit v4.8.1 (Fu et al. 2012). One representative of each cluster was further used for a similarity search on the M5nr database (https://github.com/MG-RAST/myM5NR) using the DIAMOND sequence aligner (Buchfink et al. 2015). To assess function of protein fragments, we retrieved predicted functions of best hits through Clusters of Orthologous Groups (COG) of proteins databases (Tatusov et al. 2000). There are three COG levels ranging from general to more precise function: COG1 has 4 classification levels, COG2 level has 22 classes, and COG3 level has greater than 20 324 classes (Galperin et al. 2015). The COG database is updated periodically as new genomes become available. A matrix of function abundance by samples was produced to analyze the diversity and composition profiles in R Studio v1.1.447 (R Core Team 2018).

### Alpha diversity

We first quantified alpha diversity of gene functions for all samples with the Shannon index (Shannon 1948), using the phyloseq package (McMurdie and Holmes 2013) at the COG2 and COG3 levels. The Shannon index, which includes both richness and evenness, was selected due to its reduced sensitivity to sample depth differences (Haegeman et al. 2013, Preheim et al. 2013). To ensure normality, we first transformed data with Tukey’s ladder transformation (Tukey 1977). Alpha diversity values were compared using linear mixed-effect models (lme functions) in R, with alpha diversity as the response variable, site Pd status and invasion stage at collection time as fixed effects, and site, collection years, heating, and collection methods as random effects. Significance was tested by performing 999 permutations of the data, and the resulting models were computed to generate a distribution of AIC statistics. The reference AIC (not randomized) was considered significant using a *P*-value threshold of 0.05.

### Beta diversity

Microbiome data are compositional because they are relative. The total number of counts per sample is highly variable and constrained by the maximum number of sequences reads. This induces strong dependencies among feature abundances, as an increase of one feature implies the decrease of counts for another feature so that the total number of counts does not exceed the specified sequencing depth (Calle 2019). Ignoring the compositional nature of microbiome data may lead to erroneous results. Consequently, we used the coherent Aitchison distance that respects the compositional properties of microbiome data (Aitchison 1982, Aitchison et al. 2000, Gloor et al. 2017, Calle 2019). The Aitchison distance is defined as the Euclidian distance after a centered-log transformation (CLR). However, since microbiome data contain a high number of zeros, they should be corrected prior to the centered-log transformation to ensure validity of results. We used the function transform from the microbiome package to perform the CLR transformation (Lahti and Shetty 2017). This function applies a pseudocount before taking log results. Beta diversity was then visualized with principal component analysis (PCA) using the ordinate and plot ordinate functions of the phyloseq package (McMurdie and Holmes 2013).

To test for effects of site Pd status and invasion stage, we used a distance-based redundancy analysis (db-RDA) (Legendre and Anderson 1999). This approach first decomposes distances into principal coordinates, and then applies RDA to the corresponding principal coordinates using the capscale function of the R package vegan (Oksanen et al. 2019). We also computed a partial db-RDA to better understand the influence of site Pd status and invasion stage, while controlling for potential confounding factors (Davies and Tso 1982). To select the constrained variables and to maximize degrees of freedom, we performed first a dbRDA not controlled on potential confounding factors (i.e. sites, year of collection, collection method, heating) (Table S3) and only kept significant factors for further analysis. By doing so, sites and collection years were used to constrain the partial db-RDA at COG3, whereas only collection years were used at COG2 level. Significance was tested via 999 permutations with the anova.cca function of the R package vegan. For all analyses, a *P*-value threshold of 0.05 was considered significant.

### Skin microbial functions composition

We assessed the effect of invasion stage on the composition of microbial functions down to the COG3 level using the Analysis of Composition of Microbiomes (ANCOM 2.0) (Mandal et al. 2015). ANCOM is based on the analysis of difference in pairwise log-ratios of function abundance/relative abundance, between comparison groups of interest. It is appropriate for compositional data (Gloor et al. 2017), under the assumption that a minority of features (<25%) are differentially abundant (Mandal et al. 2015). This analysis does not output a *P*-value. Instead for each function, we computed a W statistic indicating the number of significantly different pairwise log-ratios, while controlling for false discoveries. Significance level was set to 0.05 and multiple corrections were performed with the Benjamini-Hochberg procedure (Benjamini and Hochberg 1995). ANCOM analysis was conducted on unrarefied COG3-level table of the more abundant functions (>1%).

### Bipartite graph analysis

We used a bipartite graph analysis to better explore the microbial functions without *a priori* group definition. The aim is to determine how samples group and by what discriminant function. A bipartite network is a collection of nodes, grouped in two separate sets, with no interactions among nodes within a set. In the present case, the bat samples were pooled by sites and year of collection to explore relationships with functional traits at the COG3 level. We used the R package bipartite and bipartiteD3 to plot bipartite graphs and to compute modules that are aggregated sets of interacting components (Dormann et al. 2008, Terry 2018). Their defining feature is that interaction within modules is more prevalent than between modules (Newman 2003, Newman and Girvan 2004, Fortunato 2010). In other words, modules are link-rich clusters of genes in a group. In addition, we chose to compute the species specificity index, the coefficient of variation of interactions, normalized to values between 0 (low specificity) and 1 (high specificity) to target the most discriminant functions between modules (Julliard et al. 2006, Poisot et al. 2012). We performed the modules and species specificity index computation on the most abundant (>1%) functions of the eight samples pooled by year and site (177 functions).

## Results

### Alpha diversity functional profile

**Figure 1.**
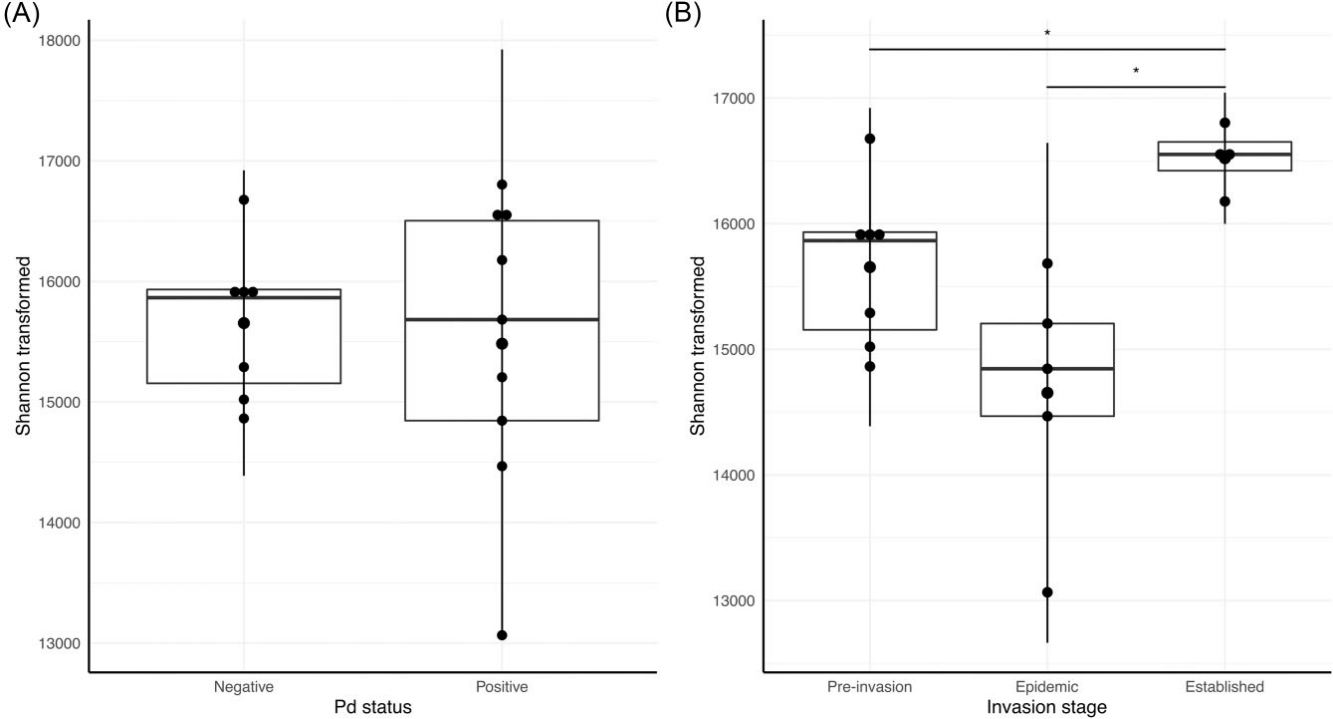
Functional Shannon diversity of skin microbiome samples transformed at the COG2 level according to (A) site Pd status and (B) invasion stage. Pairwise comparisons among invasion stages are significantly different for pre-invasion vs. established, and epidemic vs. established (Table 2).

The site Pd status model at the COG2 level (22 functions overall) was not significant (Table 2 and Fig. 1A), whereas the invasion stage model was significant (Table 2 and Fig. 1B). Mean-transformed Shannon diversity values differed by 6% (−855 ± 566) between pre-invasion and epidemic stage (Fig. 1B), but this was not significant (Table 2). Shannon diversity values from Pd-established site were significantly different from pre-invasion and epidemic stages. Alpha diversity of pre-invasion groups was significantly lower by 6% (920 ± 577) from established stage (Table 2 and Fig. 1B). Between epidemic and established stages, mean-transformed Shannon diversity was higher by 13% (1208 ± 492). The top model (based on ΔAIC) included the invasion stages: epidemic vs. established. Models at the COG3 level (2324 functions overall) were not significant (Table S2).

**Table 2.** Linear mixed models of functional alpha diversity at the COG2 levels.

Model	Model AIC	Δ AIC	Likelihood ratio	*P*-value
**Epidemic vs established**	127.940	0.000	−56.970	0.006^1^**
**Pre-invasion vs established**	150.296	22.356	−68.148	0.024^1^*
**Pre-invasion vs epidemic**	181.806	53.866	−83.903	1^1^
**Invasion stage**	225.065	97.125	−104.533	0.004**
**Site Pd status**	248.963	121.023	−117.482	0.925

1 *P*-value adjusted with Bonferroni method.

Asterisks indicate significant results.

*** $\le $ 0.001.

** $\le $ 0.01.

* $\le $ 0.05. Site, collection year, heating, and collection method were random effects. Significance tested by 999 permutations.

### Beta diversity functional profile

**Figure 2.**
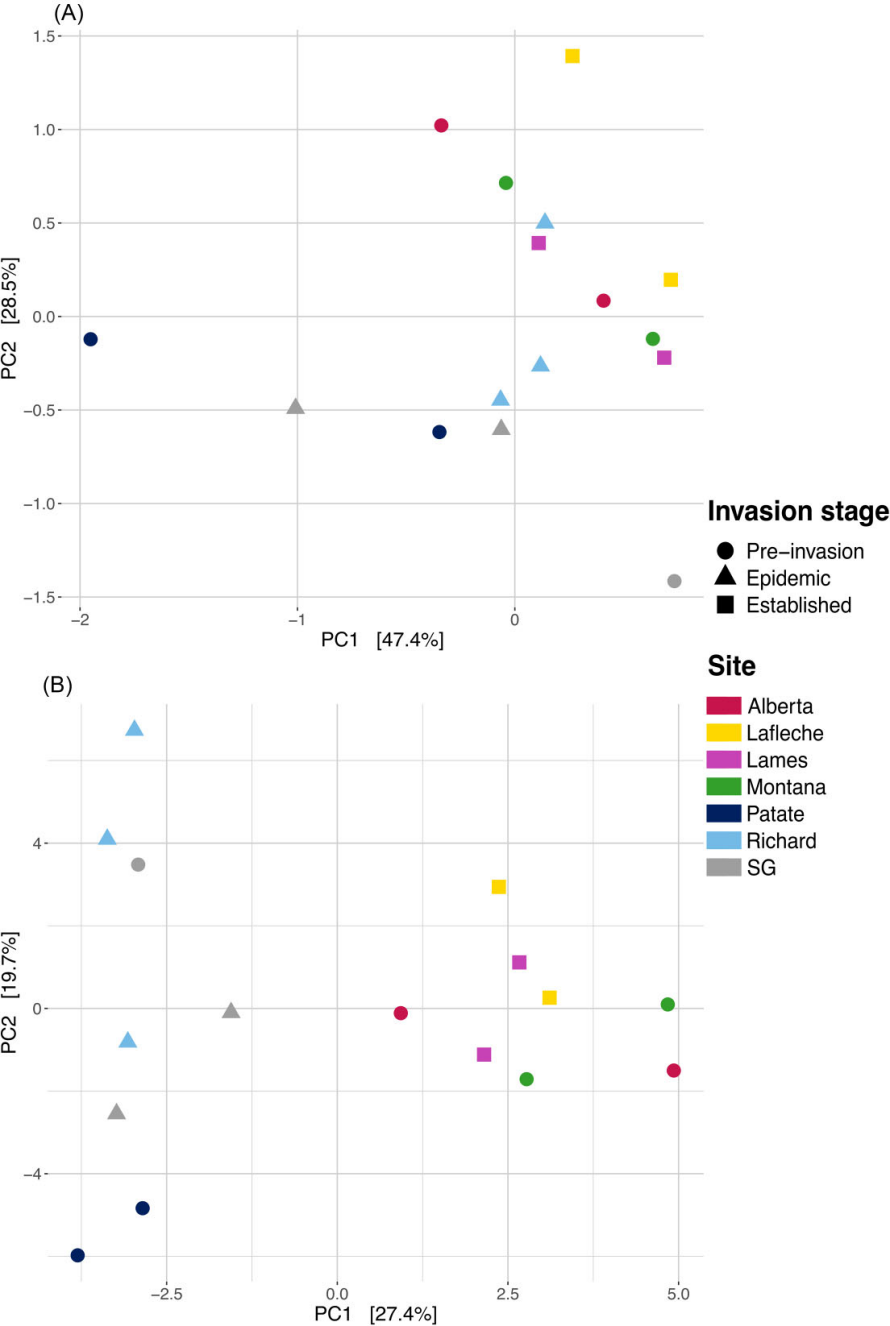
Principal component analysis of Aitchison skin microbiome functional profiles according to invasion stage and sites for (A) the COG2 level and (B) the COG3 level. Each point represents 5 bats.

Clustering according to invasion stage is not clearly defined for the COG2 level (Fig. 2A), but is much clearer for the COG3 (Fig. 2B). No models were significant at the COG2 level (Table 3). Invasion stage clusters are observable in the PCA at the COG3 level, with a clear separation on the first principal axis that explained 27.4% of variation between bats in the established stage vs. bats in the epidemic stage (Fig. 2B). However, according to the db-RDA, the confounding effect of sites was predominant and explained up to 9% of the functional variation (Table 4). These results indicate that invasion stage is nested into the site factor effect, since without site, invasion stage explained up to 8% of variation (Table 4). Even when unconstrained by site, site Pd status explained no redundant variation in the functional variation (Table 4).

**Table 3.** Db-RDA model tests of Aitchison distance of *M. lucifugus* skin microbial functions at the COG2 level.

Model formula	*F*-statistic	*P*-value	R^2^ adjusted
***∼*site Pd status**	0.42	0.81	NA
***∼*Invasion stage**	1.12	0.37	NA
***∼*site Pd status + invasion stage**	1.25	0.37	NA

Asterisks indicate significant results.

*** $\le $ 0.001.

** $\le $ 0.01.

* $\le $ 0.05.

**Table 4.** Db-RDA model tests of Aitchison distance of *M. lucifugus* skin microbial functions at the COG3 level.

Model formula	*F*-statistic	*P*-value	R^2^ adjusted
***∼* site Pd status | site, collection method, collection years**	0	NA	NA
***∼*Invasion stage | site, collection method, collection years**	0	NA	NA
***∼* site Pd status + invasion stage | site, collection method, collection years**	0	NA	NA
***∼*site | collection method, collection years**	1.50	0.05*	0.09
***∼* site Pd status | collection method, collection years**	0	NA	NA
***∼*Invasion stage | collection method, collection years**	2.31	0.007**	0.08
***∼* site Pd status + Invasion stage | collection method, collection years**	2.31	0.005**	0.08
***∼*Invasion stage + site | collection method, collection years**	1.50	0.04*	0.09

Asterisks indicate significant results.

*** $\le $ 0.001,

** $\le $ 0.01,

* $\le $ 0.05.

### ANCOM on functional profiles

**Figure 3.**
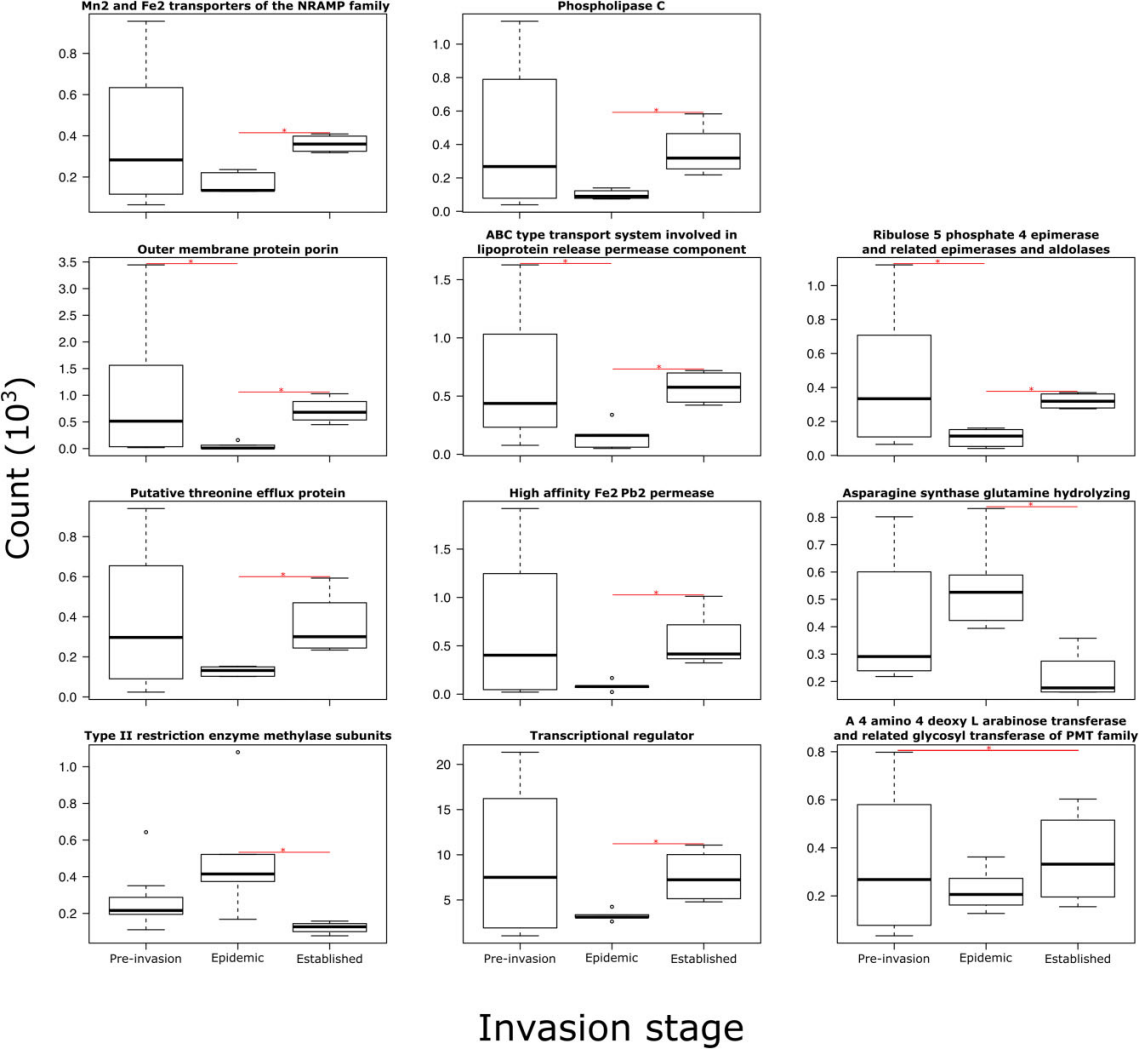
Count abundance of functions detected as significantly divergent by ANCOM according to invasion stages at 0.8 thresholds (Table S4 for W statistics). Asterisks indicate significant differences between corresponding groups.

We conducted the ANCOM analysis on the 332 more abundant COG3 functions, representing >1% of total abundance contained within the 16 pooled samples. Eleven functions differed according to invasion stage (Fig. 3 and Table S4). The epidemic samples appeared to be the most distinct. Three functions differed between the epidemic group and the pre-invasion group. Ten functions differed between the epidemic group and established group (Fig. 3). The epidemic bats exhibited the lowest abundance for the *outer membrane protein porin*, the *ABC type transport system involved in lipoprotein release permease component, ribulose 5 phosphate 4 epimerase and related epimerases and aldolases, phospholipase C, putative theorine efflux protein, high affinity Fe2 Pe2 permease, Mn2 and Fe2 transporter of the NRAMP family*, and *transcriptional regulators* compared to bats from established stage. On the contrary, the functions *asparagine synthase glutamine hydrolyzing* and *type II restriction enzyme methylase subunits* had higher abundances in the epidemic bats compared to the bats from established stage (Fig. 3). The pre-invasion and established groups appeared to have similar function abundances, except for *A 4 amino 4 deoxy L arabinose transferase and related glycosyltransferases of PMT family*, which exhibited larger functional abundances mean in the bats from the established invasion stage (Fig. 3).

### Bipartite analysis

**Figure 4.**
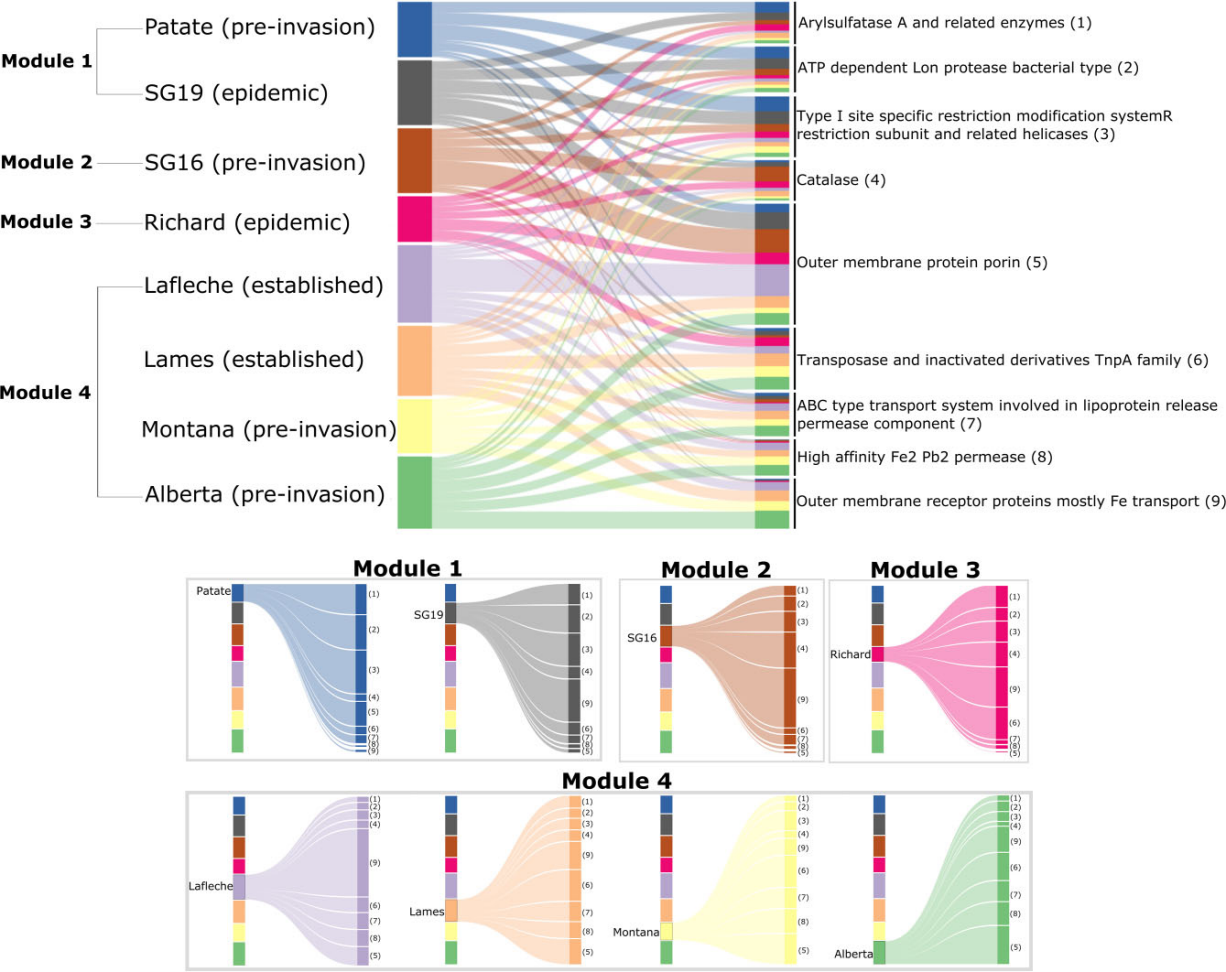
Bipartite graph associating samples pooled by sites and collection years with the more abundant functions at the COG3 level (>1%). Only the nine functions with the higher species specificity index are presented (for other functions see Table S5). The four different modules identified in the complete bipartite graph are highlighted to illustrate the relationships with the corresponding functions. Wider links indicate functions with higher relative abundance in a sample.

We explored the microbial functions without *a priori* group definition using a bipartite graph analysis of the samples pooled by site and collection year at the COG3 level. We performed the modules and species specificity index computation on the most abundant (>1%) functions of the eight pooled samples (see Table S5 for modules and the ten functions with higher species specificity by module). Out of these 177 functions, the nine functions with higher species specificity index were considered as the most discriminating between modules (Fig. 4).

The first module clusters pre-invasion bats collected from Patate with the bats in epidemic stage from St-George collected in 2019 (SG19) (Fig. 4) and is characterized by 71 functions (see Table S5). Among the nine more discriminant functions, three belong to this module, including *ATP-dependent Lon protease bacterial type, arylsulfatase A and related enzymes*, and *type I site-specific restriction modification system R restriction subunit and related helicases* (Fig. 4). Pre-invasion bats collected in 2016 (SG16) are part of a second module associated with 33 functions (Table S5), including two among the nine more discriminant functions: *catalase* and *outer membrane receptor proteins mostly Fe transport* (Fig. 4). The epidemic bats from the Richard Lake site are forming a third module characterized by 25 functions (Table S5 and Fig. 4). The last module clusters pre-invasion bats collected from sites in Montana and Alberta with colonies in the established invasion stage collected at sites Lames and Laflèche. Collectively, these four sites are characterized by 42 functions (Table S5), including four among the nine most discriminating functions: *high affinity Fe_2_ Pb_2_ permease, transposase and inactivated derivatives TnpA family, outer membrane protein porin*, and *ABC type transport system involved in lipoprotein release permease component* (Fig. 4).

## Discussion

In this study, we used shotgun metagenomics to identify the functional potential of the skin microbiome of hibernating *M. lucifugus* colonies not affected by Pd and at different stages of pathogen invasion. Our objectives were to investigate the effect of site infection status and invasion stage on the microbial functional assemblage and to understand how the microbiome could potentially influence or be influenced by fungal infection. Overall, microbial functional assemblages of bat colonies were not impacted by site Pd-positive (epidemic and established) or Pd-negative status (pre-invasion) alone. Our metagenomic results on *M. lucifugus* are consistent with Grisnik et al. (2020, 2022), who reported considerable functional and taxonomic redundancy among Pd-positive and Pd-negative individuals but in *P. subflavus* bats from epidemic and established sites. However, our results appear to differ from what was found on *M. lucifugus* taxonomic studies, which found differences in bacterial taxa among Pd-status (Lemieux-Labonté et al. 2017, Ange-Stark et al. 2023). We must stress that our results are reliable at a site/colony level and may differ from previous studies as samples from established and epidemic sites were not individually accessed for Pd. Consequently, pools in these sites were probably formed of negative and positive individuals. We suggest future studies to assess individual Pd load and metagenomes to obtain a finer-scale resolution of fungal infection intensity effect on skin microbiome functions.

Still, when we considered the disease invasion stage and not just the site positive or negative status, we did find an effect on the microbiome functional assemblages. We observed significantly lower diversity in pre-invasion and epidemic stage bats compared to the established disease stage but we did not observe a significant lower diversity in epidemic stage bats compared to pre-invasion bats. Furthermore, our results suggest that bat colonies persisting after invasion may regain Shannon diversity at higher levels than pre-invasion bats (Lemieux-Labonté et al. 2017). This could indicate an enrichment in new functions in response to the disease and/or a better evenness after invasion. Yet, this trend was only observed at higher functional levels (COG2), which suggests change in more general function groups. Consistent with these findings, our first hypothesis is not supported, as we did not observe a decrease in diversity between pre-invasion bats and epidemic stage bats, and that an increase in diversity was observed in bats at the established stage of invasion. The functional assemblage diversity differed the most between bats collected at epidemic and established invasion stages. This pattern could represent selection for a particular functional profile that could help bats survive persistent disease during the established stage or a return to a normal state similar to that occurring pre-invasion.

By analyzing functional abundance, we observed that predominant genes detected are parts of various biological processes, such as molecular functions or cellular components (UniProt 2019). We also discovered changes in predominant functions between invasion stages. Overall, the established group had a community composition similar to that of pre-invasion bats and the epidemic stage appears to be the most different. Many functions with anti-fungal potential are less abundant in epidemic stage bats. Some of these functions are involved in iron (Fe2+) and manganese (Mn2+) acquisition and transport [*Mn2 and Fe2 transporter of the NRAMP family-like protein, high affinity Fe2 Pb2 permease*, and *outer membrane receptor proteins mostly Fe*] (UniProt 2019). Iron and manganese are essential micronutrients that play a central role in infection processes because they serve as cofactors in many reactions, including many with direct and indirect roles in pathogens virulence (Gerwien et al. 2018). Hosts can also fight pathogens by deploying toxic levels of certain metals (Hood and Skaar 2012). Upregulation or downregulation of function influencing Fe2+ and Mn2+ bioavailability could influence Pd invasion (Reeder et al. 2017, Gerwien et al. 2018). We also detected less *phospholipase C*, which can be toxic and cause lysis in eukaryotic cells (Titball 1993), at the epidemic stage. However, as these functional abundances were similar between pre-invasion and Pd-established colonies, our hypothesis on the selection of anti-fungal functions following Pd establishment is not supported. The low abundance of these functions in newly infected epidemic colonies is consistent with a new hypothesis that disruption by the fungus could cause a decrease in these functions with potentially negative effects on the microbiome and bat health. Still, more work is needed to understand the implications of changes in microbiome function on disease severity and survival of bats with WNS.

Moreover, we also observed an interesting trend in pre-invasion bats. This group appears to have a wider dispersion in their functional composition abundance and diversity. Indeed, functional abundance appears more variable between pooled samples in the pre-invasion group than in the epidemic and established groups. This is also true for beta diversity analysis, where one can observe a wider dispersion for this group, with pooled samples being more different among sites. The pre-invasion group has the largest dispersion, followed by the epidemic and the established groups, supported by the significant interaction between invasion stage and site. Accordingly, sites might have a larger influence on pre-invasion microbiomes compared to the microbiome collected at epidemic and established sites. Unfortunately, this could be an artifact of the relatively few sites sampled within each category and large geographic dispersion of pre-invasion group, and we suggest further investigation to verify this trend.

Although our results are consistent with variation in effects of WNS on microbiome function depending on the stage of pathogen invasion, we must stress that shotgun metagenomics only elucidates the functional potential of putative COGs, and does not reflect what is truly expressed by the corresponding genes (Jansson and Hofmockel 2018). Transcriptomic and other meta-omics approaches will be the next step to elucidate the true skin microbial potential in the WNS context. It is also important to note that patterns we observed in this study could be site- (i.e. colony-)specific and might not reflect invasion stage, owing to microbiome differences associated with geographical/environmental variation. This site effect on function assemblage is consistent with what was found in previous taxon-based microbiota studies (Avena et al. 2016, Lemieux-Labonté et al. 2017). Thus, we could expect site-specific selection on microbiome function due to WNS. Bats might also show mechanisms of resistance or tolerance to WNS independent of the microbiome due to physiological or behavioral mechanisms such as environmental selection and physiological adaptation (Cheng et al. 2019, Langwig et al. 2017). Thus, there is a possibility that microbiome may act synergistically with other mechanisms to help mitigate Pd infection. Another potential limitation of our study is that time (i.e. year) and WNS-status (positive or negative) are confounded. However, we controlled for confounding effects such as site, collection year, and collection method in our analyses and found support for the hypothesis of an effect of invasion stage on skin functions assemblage.

This study provides evidence of changes in microbiome functional profiles that may be attributed to WNS. It represents an important step towards understanding the role of skin microbiome in wild bat colonies facing an emergent fungal pathogen. Overall, our results support that microbiome functional profiles vary among Pd invasion stages but that changes are site-specific. Such diversified responses could help explain why some colonies may be affected differently by Pd infection during the initial epidemic phase of WNS (Frick et al. 2017). Interestingly, we found that skin microbial functions of persisting bats in the established phase of WNS were similar to sites that were pre-infection, possibly indicating reestablishment of microbiomes. It is still unclear whether the skin microbiome could protect against Pd, or whether the changes we observed reflect neutral or maladaptive effects of the disease. Future studies design should use concurrent sampling from multiple positive and negative sites in different phases of WNS invasion and assess individual change in Pd load in relation to functional microbiome. Being able to have more samples and perform sequencing of individual bat samples would increase statistical power and help support or reject new hypotheses brought by this research. However, given how far the disease has spread and the range of bat species and communities now involved, this may no longer be possible, at least in the eastern half of North America. We thus suggest local authorities or researchers doing annual winter survey in western sites not affected by the disease to collect additional swabs from bats skin for microbiome study, anticipating infection, and documenting wing microbiomes throughout the infection stages. As we expect WNS to spread further, these samples will allow monitoring the microbiome throughout disease stages. This kind of sampling regime could provide better insight to understand interactions between Pd and the microbiome. Understanding the roles of the microbiome and developing new tools to better manage the disease in the future, may hinge on use of combined meta-omics approaches, and consideration of other resistance and tolerance mechanisms.

## Supplementary Material

fiae138_Supplemental_File

## Data Availability

The raw sequencing data supporting the conclusions of this article are available here: https://figshare.com/s/8c742fca1ec5e317324a. The scripts are available here: https://figshare.com/s/53545eafd27ff4a20d53.
