# Supplementary material for: Pseudogymnoascus destructans invasion stage impacts the skin microbial functions of highly vulnerable Myotis lucifugus
Source: FEMS Microbiol Ecol. 2024 Oct 14;100(11):fiae138. doi: 10.1093/femsec/fiae138 (PMC11523048; doi:10.1093/femsec/fiae138)
Supplement: fiae138_Supplemental_File [file fiae138_supplemental_file.docx]

## Supplementary files

**Table S0.** Fungal load of epidemic and established invasion stage site. All samples with values crossing baseline below 40 cycles threshold (40 C_t_) were considered positive.

| **Site** | **Collection year** | **Number of swabs collected** | **Mean Ct-Value of positive swabs** | **Positive swabs (%)** | **Site Pd-status** |
| --- | --- | --- | --- | --- | --- |
| Richard Lake | 2018 | 30 | 35,04 | 90 | Positive |
| St-George (SG) | 2019 | 19 | 31,00 | 95 | Positive |
| Lafleche | 2020 | 5 | 35,90 | 100 | Positive |
| Lames | 2020 | 5 | 38,50 | 60 | Positive |

**Table S1.** Illumina NovaSeq 6000 S4 PE150 Sequencing Shotgun library information by sample.

| **SampleID** | **Number of Reads** | **Number of Bases** | **Number of Cycles** | **Average Quality**  **(Q score)** |
| --- | --- | --- | --- | --- |
| RL18pool1 | 26803053 | 8094522006 | 318 | 36 |
| RL18pool2 | 22446671 | 6778894642 | 318 | 36 |
| Albpool1 | 20252019 | 6116109738 | 318 | 36 |
| Albpool3 | 15632117 | 4720899334 | 318 | 36 |
| Lafpool2 | 22901019 | 6916107738 | 318 | 36 |
| Lafpool3 | 22592508 | 6822937416 | 318 | 35 |
| Lamespool2 | 21542918 | 6505961236 | 318 | 35 |
| Lamespool3 | 16787323 | 5069771546 | 318 | 35 |
| Mont1pool1 | 25210427 | 7613548954 | 318 | 36 |
| Montpool3 | 17196312 | 5193286224 | 318 | 36 |
| Patpool1 | 19772503 | 5971295906 | 318 | 36 |
| Patpool3 | 7824715 | 2363063930 | 318 | 36 |
| RL18pool3 | 19740865 | 5961741230 | 318 | 36 |
| SG163pool | 15407384 | 4653029968 | 318 | 36 |
| SG192pool | 23710558 | 7160588516 | 318 | 36 |
| SG19pool1 | 22893338 | 6913788076 | 318 | 35 |

**Table S2.** Linear mixed model effect of functional Shannon diversity at COG3 levels. Only site was included as random effect because adding other random effects lead to non-convergence issue for all models. Significance was tested by 999 permutations.

| **Model** | **Model AIC** | **Likelihood ratio** | ***P*-value** |
| --- | --- | --- | --- |
| **Pd status** | 249.737 | -226.82 | 1 |
| **Invasion stage** | 433.271 | -211.64 | 1 |

Stars and bold indicate significant results. ***$\leq$0.001. **$\leq$0.01. *$\leq$0.05.

**Table S3.** Db-RDA of bat skin functional Aitchison distance at COG2 and COG3 levels for potential controlled factor. Only the significant factors are added to models in Table 3 and 4.

| **Model formula** | **COG level** | ***F* statistic** | ***P*-value** | **R^2^ adjusted** |
| --- | --- | --- | --- | --- |
| *∼*collection method | COG2 | 0.89 | 0.46 | NA |
|  | COG3 | 2.20 | **0.02*** | 0.074 |
| *∼*heated | COG2 | 0.39 | 0.81 | NA |
|  | COG3 | 1.62 | 0.07 | NA |
| *∼*site | COG2 | 1.62 | 0.09 | NA |
|  | COG3 | 2.35 | **1e-0.4***** | 0.35 |
| *∼*collection years | COG2 | 1.40 | 0.12 | NA |
|  | COG3 | 1.62 | **1e-0.3***** | 0.14 |

Stars and bold indicate significant results. ***$\leq$0.001. **$\leq$0.01. *$\leq$0.05. COG2 total response matrix inertia = 3.5345. COG3 total response matrix inertia = 1641.0519.

**Table S4.** ANCOM W statistic results of abundant COG3 function while adjusting for sites, collection years, heated, and collection method, for invasion stage group. Only significantly different abundant functions at threshold 0.8 are presented.

| **Pairwise comparison** | **Functions COG3** | **W stat** |
| --- | --- | --- |
| 1-year vs. 10-year | Outer membrane protein porin | 332 |
|  | ABC type transport system involved in lipoprotein release permease component | 323 |
|  | Ribulose 5 phosphate 4 epimerase and related epimerases and aldolases | 312 |
|  | Phospholipase C | 297 |
|  | Putative threonine efflux protein | 293 |
|  | High affinity Fe2 Pb2 permease | 287 |
|  | Asparagine synthase glutamine hydrolyzing | 280 |
|  | Mn2 and Fe2 transporters of the NRAMP family | 278 |
|  | Type II restriction enzyme methylase subunits | 275 |
|  | Transcriptional regulator | 269 |
| 0-year vs. 1-year | Outer membrane protein porin | 332 |
|  | ABC type transport system involved in lipoprotein release permease component | 322 |
|  | Ribulose 5 phosphate 4 epimerase and related epimerases and aldolases | 315 |
| 0-year vs. 10-year | A 4 amino 4 deoxy L arabinose transferase and related glycosyltransferases of PMT family | 278 |

**Table S5.** Module list and Species specificity index of COG3 functional genes on most abundant predicted genes (>1%). The ten functions with the highest Species specificity index are presented for each module.

| **Module** | **COG3 functions** | **Species specificity** |
| --- | --- | --- |
| Module 1 | ATP dependent Lon protease bacterial type | 0.23 |
|  | Arylsulfatase A and related enzymes | 0.22 |
|  | Type I site specific restriction modification system R restriction subunit and related helicases | 0.20 |
|  | Inorganic pyrophosphatase | 0.18 |
|  | Response regulator containing CheY like receiver AAA type ATPase and DNA binding domains | 0.16 |
|  | Transposase and inactivated derivatives | 0.15 |
|  | Asparagine synthase glutamine hydrolyzing | 0.15 |
|  | Mismatch repair ATPase MutS family | 0.14 |
|  | Type I restriction modification system methyltransferase subunit | 0.14 |
|  | DNA directed RNA polymerase sigma subunit sigma70 sigma32 | 0.13 |
| Module 2 | Catalase | 0.29 |
|  | Outer membrane receptor proteins mostly Fe transport | 0.21 |
|  | Subtilisin like serine proteases | 0.18 |
|  | Large extracellular alpha helical protein | 0.16 |
|  | A 1 4 alpha glucan branching enzyme | 0.16 |
|  | Long chain acyl CoA synthetases AMP forming | 0.16 |
|  | ABC type sugar transport systems permease components | 0.15 |
|  | Predicted metal dependent hydrolase with the TIM barrel fold | 0.15 |
|  | Lhr like helicases | 0.14 |
|  | Predicted dehydrogenases and related proteins | 0.13 |
| Module 3 | ABC type transport system involved in resistance to organic solvents periplasmic component | 0.16 |
|  | ABC type sugar transport system permease component | 0.15 |
|  | Site specific recombinases DNA invertase Pin homologs | 0.14 |
|  | Cysteine synthase | 0.11 |
|  | Acyl dehydratase | 0.11 |
|  | DNA segregation ATPase FtsK SpoIIIE and related proteins | 0.09 |
|  | HrpA like helicases | 0.09 |
|  | Superfamily I DNA and RNA helicases | 0.09 |
|  | ABC type sugar transport system periplasmic component | 0.07 |
|  | Phosphomannomutase | 0.07 |
| Module 4 | Outer membrane protein porin | 0.37 |
|  | High affinity Fe2 Pb2 permease | 0.31 |
|  | Transposase and inactivated derivatives TnpA family | 0.20 |
|  | ABC type transport system involved in lipoprotein release permease component | 0.20 |
|  | Arabinose efflux permease | 0.19 |
|  | ABC type uncharacterized transport system permease component | 0.18 |
|  | Transcriptional regulators | 0.18 |
|  | RecB family exonuclease | 0.16 |
|  | ABC type nitrate sulfonate bicarbonate transport systems periplasmic components | 0.16 |
|  | Membrane fusion protein | 0.16 |
